# Heterogeneity and overlap in the continuum of linguistic profile of logopenic and semantic variants of primary progressive aphasia: a Profile Analysis based on Multidimensional Scaling study

**DOI:** 10.1186/s13195-024-01403-0

**Published:** 2024-03-07

**Authors:** Gaia Chiara Santi, Francesca Conca, Valentina Esposito, Cristina Polito, Silvia Paola Caminiti, Cecilia Boccalini, Carmen Morinelli, Valentina Berti, Salvatore Mazzeo, Valentina Bessi, Alessandra Marcone, Sandro Iannaccone, Se-Kang Kim, Sandro Sorbi, Daniela Perani, Stefano F. Cappa, Eleonora Catricalà

**Affiliations:** 1 IUSS Cognitive Neuroscience (ICoN) Center, Scuola Universitaria Superiore IUSS, Pavia, Italy; 2 IRCCS Mondino Foundation, Pavia, Italy, Pavia, Italy; 3 IRCCS Fondazione Don Carlo Gnocchi, Florence, Italy; 4 Department of Brain and Behavioural Sciences, University of Pavia, Pavia, Italy; 5 Vita-Salute San Raffaele University, Milan, Italy; 6 Research and Innovation Centre for Dementia-CRIDEM, Azienda Ospedaliero-Universitaria Careggi, Florence, Italy; 7 Department of Biomedical Experimental and Clinical Sciences, University of Florence, Florence, Italy; 8 Department of Neuroscience, Psychology, Drug Research and Child Health, University of Florence, Florence, Italy; 9 Department of Rehabilitation and Functional Recovery, San Raffaele Hospital, Milan, Italy; 10 Department of Paediatrics, Baylor College of Medicine, Houston, TX USA; 11 Nuclear Medicine Unit, San Raffaele Hospital, Milan, Italy

**Keywords:** Logopenic PPA, Semantic PPA, Lexico-semantics, Phonology, Verbal working memory, Profile Analysis via Multidimensional Scaling, FDG-PET

## Abstract

**Background:**

Primary progressive aphasia (PPA) diagnostic criteria underestimate the complex presentation of semantic (sv) and logopenic (lv) variants, in which symptoms partially overlap, and mixed clinical presentation (mixed-PPA) and heterogenous profile (lvPPA +) are frequent. Conceptualization of similarities and differences of these clinical conditions is still scarce.

**Methods:**

Lexical, semantic, phonological, and working memory errors from nine language tasks of sixty-seven PPA were analyzed using Profile Analysis based on Multidimensional Scaling, which allowed us to create a distributed representation of patients’ linguistic performance in a shared space. Patients had been studied with [^18^F] FDG-PET. Correlations were performed between metabolic and behavioral data.

**Results:**

Patients’ profiles were distributed across a continuum. All PPA, but two, presented a lexical retrieval impairment, in terms of reduced production of verbs and nouns. svPPA patients occupied a fairly clumped space along the continuum, showing a preponderant semantic deficit, which correlated to fusiform gyrus hypometabolism, while only few presented working memory deficits. Adjacently, lvPPA + presented a semantic impairment combined with phonological deficits, which correlated with metabolism in the anterior fusiform gyrus and posterior middle temporal gyrus. Starting from the shared phonological deficit side, a large portion of the space was occupied by all lvPPA, showing a combination of phonological, lexical, and working memory deficits, with the latter correlating with posterior temporo-parietal hypometabolism. Mixed PPA did not show unique profile, distributing across the space.

**Discussion:**

Different clinical PPA entities exist but overlaps are frequent. Identifying shared and unique clinical markers is critical for research and clinical practice. Further research is needed to identify the role of genetic and pathological factors in such distribution, including also higher sample size of less represented groups.

**Supplementary Information:**

The online version contains supplementary material available at 10.1186/s13195-024-01403-0.

## Background

Current criteria for diagnosis of logopenic/phonological variant of primary progressive aphasia (PPA) (lvPPA) [1] describe core (impaired single word retrieval and sentence repetition) and secondary (phonological errors, spared semantic knowledge, grammar, and motor speech) features. Impairments of auditory-verbal working memory (WM), word retrieval deficit, and phonological errors are associated with the dysfunction of the temporo-parietal junction [2].

A considerable heterogeneity in the clinical presentation of this PPA variant has been described [3], with patients showing only the cardinal features [4], or presenting a conjunction of semantic deficit and agrammatism. Different authors already stressed the need for new/updated diagnostic criteria, that can account for the complexity of lvPPA presentation [5].

The overlap between lvPPA and the non-fluent/agrammatic variant (nf/avPPA) has been extensively studied [6–8]. The distinction between non-fluent and logopenic features in connected speech production depends on a careful qualitative analysis, based on the distinction between motor speech disorder, resulting in articulatory distortions and dysprosodia, and slow, hesitant (dysfluent) production. Phonological errors are typical of the lvPPA but may be present also in nf/avPPA patients, and the distinction with articulatory errors may be difficult [2, 9–11].

Similarly, deficits at auditory-verbal WM have been reported in lvPPA and nf/avPPA [12, 13]. Generally, lvPPA performance is characterized by deficits in repetition and comprehension of long sentences, associated with atrophy in temporo-parietal regions. Those deficits are partially overlapping with ones reported in nf/avPPA patients, who are impaired in word and sentence repetition [3], and sentence comprehension tasks [14].

An overlap with the semantic variant is also possible when assessing semantic knowledge, which is typically impaired in svPPA [1], in association with severe atrophy of the anterior temporal lobe. Despite the svPPA variant being relatively homogeneous at clinical, imaging, and pathological levels [11], clinical evidence revealed a possible overlap between lvPPA and svPPA linguistic profiles [15]. The presence of the semantic impairment in some lvPPA patients has been demonstrated using naming, word comprehension, and semantic association tasks, and it has been associated with the extension of atrophy from the left inferior and medial temporal lobe to the anterior temporal lobe, including the temporal pole and the fusiform cortex [16–18]. Similarly, impairment of single-word comprehension and category fluency has been reported in lvPPA patients with positive Alzheimer’s biomarkers, who showed hypometabolism extending to anterior temporal regions [4]. lvPPA patients with GRN mutations presented high variability in cognitive performance [19, 20]. Defective single-word comprehension, mixed deep/phonological dyslexia, associated with the spreading of atrophy from posterior to anterior temporal regions, are reported in those subjects [21]. Generally, this progression pattern is associated with an atypical logopenic profile with semantic impairment in more advanced stages of the disease. Conversely, svPPA shows with lexical retrieval deficit in naming task, especially in the early phase of the disease [22, 23]. Lexical and semantic impairments may occur in both svPPA and lvPPA, probably at different stages of disease [24, 25], and at different levels of severity, leading to a non-trivial behavioral overlap. In addition, mild impairment in auditory, verbal WM has been described in svPPA, as well as errors in sentence repetition and comprehension task [24] and in digit span [12]. While the severity of WM deficit might be related to differences in atrophic pattern [12], the stage of the disease could play a role in the progression of WM deficit, contributing to the emergence of overlaying profiles.

Despite evidence in support of similarities between semantic and logopenic variants, a comprehensive characterization of such complex presentations is lacking. Graded and multidimensional approaches are now frequently used in neurodegenerative research, as they overcome categorical organization and capture between and within-group variation [11].

## Methods

Consistently, the goal of the present study was to profile patients with a diagnosis of lvPPA, svPPA, and mixed lv/sv PPA, using a linguistic multidimensional space based on the relevant linguistic domains. The application of Profile Analysis Based on Multidimensional space (PAMS), in particular, permits to identify a set of impaired and spared abilities and to bring insight into the part of the space that each patient occupies, visualizing overlaps and differences in the semantic, and logopenic variants and their mixed clinical presentation. Differently from other cluster techniques [26], in fact, PAMS do not impose constraints by categorizing subjects into mutually exclusive groups. It is thus extremely valuable when dealing with a population including heterogeneous cases with blurred distinctions, in which similarities and differences between patients are particularly subtle.

Moreover, the neural correlates of the resulting relevant linguistic measures were correlated with FDG-PET imaging data.

### Participant

PPA is a rare neurodegenerative condition, with a global prevalence ranging from 2.3/100.000 to 44.1/100.00 according to the different underlying pathology [27]. Therefore, a total of sixty-seven right-handed PPA patients were retrospectively enrolled in the period between 2015 and 2020 for the present study. Data of patients were collected from the databases of the Neurology Unit I of Careggi University Hospital of Florence (61 patients) and of the Clinical Neuroscience Department, San Raffaele Hospital of Milan and University Vita-Salute San Raffaele of Milan (6 patients). All clinical diagnoses were made by expert neurologists (AM, SI, SS, SM, SFC, VB) on the basis of current clinical diagnostic criteria [1]. The sample included lvPPA + who, following Saracino and colleagues [20], presented, in conjunction with predominant retrieval and repetition impairments, mild semantic deficits, characterized by semantic errors in naming tasks, semantic association deficits, and reduced word comprehension ability, such as subtle additional linguistic deficits not fitting criteria for mixed PPA. Mixed PPA instead met criteria for both the semantic and logopenic variants, i.e., neither of the characteristic features of svPPA or lvPPA were predominant, but both were present [20]. Twenty svPPA, 19 lvPPA, 23 lvPPA + , and 5 mixed-PPA were thus included in the study. Additional biomarker evidence (Amyloid PET) was available for 18 patients, but was not used for syndromic classification.

#### Neuropsychological assessment

All patients underwent a comprehensive neuropsychological assessment, evaluating verbal and visuo-spatial short and long-term memory, attentional and executive functions, visuo-spatial functions, praxis, and language (see Supplementary Table 1, Additional File 1).

Language assessment was based on SAND battery, which included 9 subtests [28]. The number of semantic (SEM), phonological (PHON), and working memory (WM) errors was derived from the appropriate subtests, as in Catricalà et al. [24, 29], and reported in Table 1. Scores were then normalized for the maximum possible value (or for measures without a maximum score for the maximum values obtained by patients). In order to give the same direction to all scores, the inaccuracy rate (1 — single subject score) for each error of each domain was calculated, with the exclusion of the number of nouns and verbs in the picture description.

**Table 1:** Description of the scores considered for semantic, phonologic, and working memory domains of error

Error classification	
Type of error	SAND task
SEM	Semantic errors in naming
	Errors in word comprehension
	Stress errors in reading
	Errors in semantic association
	PD: N° nouns/N° words
	PD: N° verbs/N° words
	PD: N° self-corrected sequences/N° words
	PD: N° semantic errors/N° words
PHON	Phonological errors in naming
	Phonological errors in word repetition
	Phonological errors in non-word repetition
	Phonological errors in sentence repetition
	Phonological errors in word reading
	Phonological errors in non-word reading
	PD: N° phonological errors/N° words
WM	Working memory errors in long sentences in sentence comprehension
	Working memory errors in sentence repetition

*PD* picture description, *N°* number, *SEM* semantic, *PHON* phonologic, *WM* working memory

### Demographic and clinical data

We compared demographic data between groups using the Kruskal–Wallis *H* test and Bonferroni’s correction for continuous variables; meanwhile, the chi-square test was adopted for categorical variables.

### Profile Analysis based on Multidimensional Scaling (PAMS)

PAMS analysis is a technique based on a multidimensional scaling approach (for a full description of the technique see [30]), allowing to estimate from the data the “core profiles”, which are conceptualized as the prototypical response pattern among participants, with peaks and valleys representing, respectively, preserved, and impaired abilities. Each core profile may be considered both at *direct* and at *inverse* direction. PAMS also provides the extent to which each subject fits to each core latent profile in terms of weights.

Qualitative behavioral data derived from SAND were included in PAMS. The appropriate dimensionality of PAMS was selected according to the index of fit values (stress ≤ 0.05, [31]) and considering the clinical coherence of data. Bootstrapping analysis (*n* = 2000) was performed on derived core profiles, and confidence intervals (CI) at the 95th percentile were estimated. Core profiles are expected to be orthogonal, but to be sure of this we estimated their collinearity, in terms of *R*^2^, among latent profiles regressing each core profile against the others. We assessed the amount of variance accounted by the full model for the sixty-seven participants. Values for each variable of each core profile were converted into *z*-score and, for each core profile, the two features with the higher and lower values were considered as relevant peaks and valleys, respectively. The proximity between each single subject and each core profile was assessed using weight values. A high positive weight indicates that the subject’s performance is closely related to the direct core profile, whereas a high negative weight indicates that the subject’s performance is closer to the inverse direction of a core profile. Weight values close to zero suggest no closeness to a core profile [32]. Proximity judgment of single patient performance to one or more core profiles was based on the observation of the absolute value of weights. We attributed a patient to a profile if the respective weight had a value greater than |0.1|. Usually, the first core profile represents the mean performance of subjects [30], so we expected that most patients would have higher weight values for this profile. We then decided to consider also the second higher weight in order to better describe the linguistic profile of patients. Affiliation to a direct or inverse profile was attributed according to weight’s sign (i.e., positive, or negative). We plotted values of picks and valleys derived from PAMS profiles in order to visualize the distribution of patients across them. As an exploratory analysis, we compared subjects belonging to the different profiles for demographic data to check for their possible impact, and tests emerged as significant from PAMS profiles; results are reported in Supplementary Table 2 of the Additional File 1.

### FDG-PET acquisition

Sixty-six out of the 67 patients underwent an FDG-PET scan acquisition. All FDG-PET scans were performed within 3 months of neuropsychological assessment. According to the EANM guidelines [33] patients were tested for blood sugar levels to be lower than 120 mg/dl. Then subjects were injected with 185 MBq of [18F]-FDG via a venous cannula. After the injection, patients were left in a dim, quiet room and told to keep their eyes closed. Patients were scanned using General Electric’s Medical Systems Discovery-STE scanner (in Milan) or Gemini TF PET/CT (Philips Medical Systems) (in Florence). A multi-center collection of data was already employed in a previous study of our group [24, 34]. Considering the multi-center nature of the study (Florence/Milan), all PET images were corrected for photon attenuation, scatter, and radioactive decay and reconstructed using an Order Subset Expectation Maximization algorithm. First, each patient’s PET image was analyzed using a standardized single-subject procedure based on statistical parametric mapping (SPM), involving image: (1) spatial normalization by means of a dementia-specific template; (2) smoothing with an isotropic 3D Gaussian kernel with a FWHM of 8 mm in each direction; and (3) intensity-normalization with a whole-brain reference region (see [35]). Then, each patient scan was assessed for relative hypometabolism based on statistical parametric mapping (SPM) procedures including comparison with a large image database of FDG-PET scans of healthy controls (HC). The well-selected HC dataset is presently used in research and clinical settings [36].

#### Selection of the regions of interest

Relevant regions of interest (ROIs) were selected considering the existing literature investigating the cerebral regions involved in PPA, as well as the processes of interest in the current study, namely lexical-semantics, phonology, and verbal working memory [14, 34, 37, 38], see Table 2.

**Table 2:** List of the nine selected ROIs and the respective MNI coordinates from AAL Atlas

Regions of Interest				
**Laterality**	**Label**	***X***	***Y***	***Z***
Left	Inferior frontal gyrus (triangularis) (IFG)	− 45.58	29.91	13.99
Left	Middle/superior frontal gyrus	− 33.43	32.73	35.46
Left	Posterior fusiform gyrus	− 31.16	− 40.3	− 20.23
Left	Anterior fusiform gyrus	− 31.16	− 40.3	− 20.23
Left	Inferior parietal lobule (PL)	− 42.8	− 45.82	46.74
Left	Supra marginal gyrus	− 55.79	− 33.64	30.45
Left	Posterior middle temporal gyrus (MTG)	− 55.52	− 33.8	− 2.2
Left	Anterior superior temporal gyrus (STG)	− 53.16	− 20.68	7.13
Left	Posterior superior temporal gyrus (STG)	− 53.16	− 20.68	7.13

*IFG* inferior frontal gyrus, *PL* parietal lobule, *MTG* middle temporal gyrus, *STG* superior temporal gyrus

Nine ROIs, located in the left hemisphere, were created from the Automated Anatomical Labelling (AAL) atlas [39]. Anterior and posterior sections of the fusiform and superior temporal gyri were defined in line with the boundaries in the AAL atlas and according to Visser et al. [40], i.e., the MNI coordinates of posterior sections extended from *y* =  − 24 to − 60 for fusiform, *y* =  − 24 to − 58 for inferior, and *y* =  − 19 to − 51 for the superior temporal areas.

A series of linear regression analyses was performed to investigate the metabolism of which ROIs predicted the error type reported as relevant in determining the core profiles, in the whole sample. In order to individuate the ROIs to include in the models, correlation analyses between patients’ scores identified as relevant peaks and valleys of each core profile and metabolic values of ROIs were performed, on the whole sample.

## Results

### Demographic and clinical data

Demographical and clinical data of patients are reported in Table 3. There were no significant differences for gender, age, and education between variants (i.e., svPPA, lvPPA, lvPPA + , and mixed-PPA) (all *p* values > 0.25). Significant differences were found between svPPA and lvPPA + in MMSE and between lvPPA and lvPPA + in disease duration.

**Table 3:** Demographical and clinical data of patients’ groups

Demographic and clinical data				
Patients group	**svPPA (20)**	**lvPPA (19)**	**lvPPA + (23)**	**Mixed-PPA (5)**
Gender, M|F	11|9	10|9	10|13	2|3
Age, years, mean (SD)	67.95 (7.94)	68.37 (6.13)	71.74 (7.53)	67.60 (6.22)
Education, years, mean (SD)	11.50 (4.28)	11.11 (3.19)	11.70 (4.80)	11.40 (4.66)
Disease duration, months, mean (SD)	27.95 (15.8)	20.63 (8.64)^a^	32.00 (13.95)^a^	24.00 (20.78)
MMSE, Corrected score, mean (SD)	22.56 (5.46)^a^	20.98 (5.21)	16.81 (6.30)^a^	23.05 (2.73)

*svPPA* semantic variant of primary progressive aphasia, *lvPPA* logopenic variant of primary progressive aphasia meeting canonical criteria, *lvPPA* + logopenic variant of primary progressive aphasia with additional symptoms, *mixed-PPA* mixed variant of primary progressive aphasia, all subjects are semantic-logopenic, *SD* standard deviation, *MMSE* Mini-Mental State Examination

^a^Significant difference after Bonferroni’s correction

### Profile Analysis based on Multidimensional Scaling (PAMS)

**Fig. 1:**
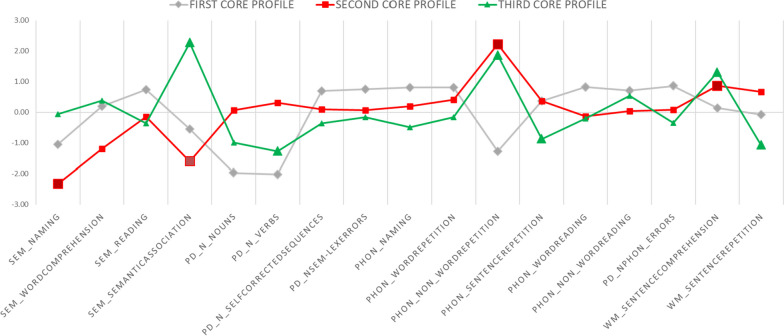
The three core profiles derived by PAMS. Bold squares and triangles represent the measures used to characterize the second and third profiles, see text for details; *SEM*, semantic errors; *PHON*, phonological errors; *WM*, working memory errors; *PD*, picture description

Three core profiles (see Fig. 1) explained 83% of the response pattern variance among subjects, with a good fit index (stress = 0.017). The bootstrapping 95% CI was between 0.010 and 0.026, where the observed stress value of 0.017 was included, confirming the statistical stability of the stress. Collinearity accounted for less than 1% of each core profile (all *R*^2^ < 0.001).

#### First core profile

The number of nouns and verbs from the picture description are the main valleys, which may represent lexical abilities impairment. The number of phonological errors in the words repetition and in the picture description task are the main peaks of this profile (1D), which may represent phonological abilities impairment.

#### Second core profile

The number of semantic errors in naming and the total errors in the semantic association task were the valleys of this profile, while phonological errors in non-words repetition and working memory errors in sentence comprehension were the peaks. The direct direction (2D) of this profile is therefore characterized by an impairment at the semantic domain and by a good performance on phonology and working memory, the inverse profile (2I) presented instead the opposite pattern.

#### Third core profile

The number of verbs generated in the picture description and the number of working memory errors reported in the sentence repetition task were the valleys identified for the third profile. The peaks were represented by the total number of errors in the semantic association task, and the phonologic errors in non-words repetition. The direct direction of the third profile (3D) was then characterized by a reduced production of verbs in picture description and by an impairment at working memory as assessed using sentence repetition, and by preserved semantics, and phonology in non-words repetition. The inverse profile (3I) presented instead the opposite pattern.

#### Patient distribution across core profiles

Considering individual weights, we identified similarities between each patient and the three core profiles. Sixty-five out of sixty-seven patients belonged to the 1D, 15 to the 2D, 17 to the 2I, 19 to the 3D, and 16 to the 3I. No patient belonged to the inverse first profile. We individuate the following combinations of profiles:

#### First profile

Two subjects (1 svPPA, 1 lvPPA) belonged only to the 1D, suggesting a lexical deficit.

#### First and direct second profiles

Fourteen patients (12 svPPA, 1 lvPPA, 1 lvPPA +) showed higher similarity with the 1D and 2D profiles presenting with a typical lexical-semantic impairment, namely reduced production of verbs and nouns, semantic errors in naming and semantic association tasks.

#### First and inverse second profiles

Sixteen patients (9 lvPPA, 5 lvPPA + , and 2 mixed PPA) showed a high similarity with the 1D and the 2I profiles, with a performance characterized by lexical deficits, and impaired phonology and working memory, presenting deficits in non-word repetition and sentence comprehension.

#### First and direct third profiles

Eighteen patients (5 svPPA, 8 lvPPA, 4 lvPPA + , and 1 PPA mixed) were highly similar to the 1D and 3D profiles, showing pronounced lexical deficit and sentence repetition impairment, involving working memory disruption.

#### First and inverse third profiles

Fifteen patients (1 svPPA, 13 lvPPA + , and 1 mixed PPA) showed high similarity with the 1D and 3I profiles, presenting with a mixed impairment involving semantic and phonology competencies, as described by reduced production of verbs and nouns in oral production, semantic errors in semantic association task and phonological errors in non-words repetition.

#### Direct second and direct third profiles

One patient (1 svPPA) showed a higher correlation with the 2D and 3D profiles, presenting with semantic errors in naming and association tasks and reduced production of verbs in picture description and working memory impairment in sentence repetition.

#### Inverse second and inverse third profiles

One patient (1 mixed PPA) showed a higher correlation with the 2I and 3I profiles, namely showing a performance characterized by phonological errors in non-word repetition, working memory errors in sentence comprehension, and semantic errors in semantic association tasks.

As 9 patients (namely 7 svPPA, 1 lvPPA, and 1 lvPPA +) fell in unexpected profiles, i.e. not within the profile populated by the majority of patients with the same variant, in these cases we looked for the possible presence of different characteristics in terms of demographic data (age, education, and disease duration). Using the test of Crawford and Garthwaite [41], we compared each single patient falling in an unexpected profile (namely, 1 svPPA falling in the 3I profile, 1 svPPA falling in both 2D and 3D profiles; 1 lvPPA falling in 2D profile, and 1 lvPPA + falling in 2D profile) with patients with the same a priori defined variant (namely all sv-PPA patients of the 2D profile, all lvPPA patients of the 2I and 2D profiles, and lvPPA + patients in 3I profile, respectively). Only the lvPPA patient falling in the 2D profile showed a longer disease duration with respect to the other lvPPA patients (of the 2I and 2D profiles). In addition, the 5 svPPA patients falling in the 3D profile were compared with the other svPPA patients of the 2D profile using the Mann–Whitney test, showing no differences; see Supplementary Table 3, in Additional File 1.

**Fig. 2:**
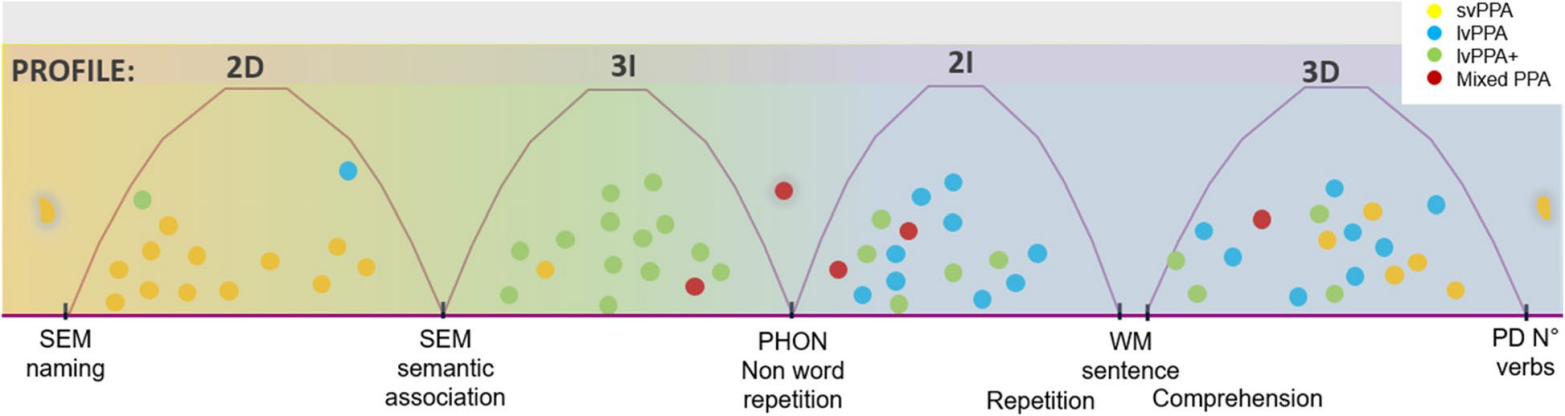
The semantic-logopenic continuum. A schematic representation of the space where all patients are distributed across the PAMS profiles and the language features. Curves represent the profiles, and relevant qualitative features for each profile are represented on the abscissa delimiting the area of each profile. Colored circles represent PPA patients. The position of each patient within the profile is determined on the basis of the feature more compromised, with patients nearer to the most impaired feature of the respective profile. Colored areas of the continuum indicate the color of the type of patients mainly populating the area. For example, the yellow area, characterized by the 2D profile, is mostly populated by the yellow circles representing svPPA patients

As 65 out of 67 patients belonged at least to the first profile, the distribution of patients across core profiles was plotted into a bi-dimensional space, representing the second and third profiles (Fig. 2).

### FDG-PET results

Linear regressions were computed and disease duration and MMSE corrected score were entered as covariates. Covariates, however, did not significantly predict any of the errors (all *p* values > 0.05). The number of nouns produced in the picture description task was significantly predicted by the metabolic values in the posterior MTG (F(1,52) = 6.038, *p* = 0.017), posterior STG (F(1,52) = 3.999, *p* = 0.050) and anterior STG (F(1,52) = 7.654, *p* = 0.008). Semantic errors in naming task were predicted by the metabolism of the posterior fusiform gyrus (F(1,52) = 6.527, *p* = 0.013) and anterior fusiform gyrus (F(1,52) = 32.646, *p* < 0.001). Phonological errors in non-word repetition were predicted by metabolic values in the anterior fusiform gyrus (F(1,52) = 6.196, *p* = 0.016) and posterior MTG (F(1,52) = 4.705, *p* = 0.034). Lastly, working memory errors in a sentence comprehension task were predicted by the activity of the middle/superior frontal gyrus (F(1,52) = 4.156, *p* = 0.046), the inferior PL (F(1,52) = 5.039, *p* = 0.028), and the posterior MTG (F(1,52) = 7.038, *p* = 0.010).

## Discussion

To characterize the logopenic and semantic linguistic profile along a continuum, we used a multidimensional technique, allowing for a flexible characterization of patients.

We identified different combinations of impaired/spared linguistic abilities in terms of 3 profiles, characterized by seven features, which described all patients along a semantic-logopenic continuum. This was easily achieved because profiles shared certain features, preventing a clear demarcation (Fig. 2).

The common feature among most patients was a lexical impairment, captured by the first (1D) profile, and related to the reduced number of produced nouns and verbs. The continuum started with the (2D) profile marked by semantic features, namely semantic errors in naming and association task. Consistently, this area (yellow, Fig. 2) was primarily populated by svPPA patients. This profile shared a semantic feature, the association task, with 3I profile, additionally marked by a phonological deficit, specifically by phonological errors in non-word repetition. 3I’s area (green, Fig. 2) was mostly populated by lvPPA + patients. The phonological deficit was shared with a third area of the continuum (blue, Fig. 2), populated by all, but one, lvPPA patients. This area included two different profiles, 2I and 3D, marked by cardinal and secondary features for lvPPA diagnosis [1]. Phonological deficit and WM impairment in sentence comprehension characterized profile 2I; while the profile 3D shared WM impairment assessed with the repetition task and presented a marked lexical impairment for verbs production.

### Distribution of PPA patients across language domains

*Lexical impairments*, described as reduced production of nouns and verbs, characterized the 1D profile, at which all patients, but two, belonged, reflecting the typical anomic pattern of svPPA and lvPPA. Anomic production, elicited using connected speech [42, 43], was ascribed to different causes: the loss of semantic knowledge in svPPA and the struggle to access the lexical representation of words in lvPPA [1]. Also, mixed PPA and lvPPA + presented lexical deficits [20, 44, 45] although the cause remains undefined.

Metabolism in posterior MTG, posterior and anterior STG, which was previously associated to lexical-retrieval in healthy participants [46] and PPA [47], predicted the number of nouns. Posterior temporal regions have been linked to semantic processing and semantic errors in stroke [48], and PPA [49] patients. These regions are responsible for the representation of phonologic forms, relevant for speech production and verbal WM tasks [50]. While the posterior MTG plays a critical role in different linguistic processes, i.e., phonological, lexico-semantic, and syntax [51, 52] (see also below), the posterior STG is primarily linked to phonological aspects [53], and is correlated with WM deficit [9], and phonological errors in lvPPA [16]. Conversely, the anterior part of STG is involved in verbal semantics [40], due to its connection with auditory and language-related networks [54], leading to lexico-retrieval impairment, and reduced production of verbs and nouns, especially in svPPA [43].

Overall, this metabolic correlation pattern accounted for all various mechanisms underlying anomic patterns in svPPA, lvPPA, and their mixed manifestations.

The *semantic* features emerging in the 2D profile, characterized by semantic errors in association and naming tasks, represented the majority of svPPA patients. These results speak in favor of the homogeneity of the semantic variant, recognizable as a lump in the continuum, apart from few exceptions, i.e., see below cases linked to 3D profile.

Semantic errors in picture naming were associated with hypometabolism in the anterior and posterior fusiform gyrus. Fusiform gyrus is a core component of conceptual knowledge circuitry [34]. Its posterior section is linked to the visual elaboration and color processing [55], while the anterior portion is implied in high-level semantic processing, integrating multiple sources of information [56, 57]. Fusiform gyrus was linked to lexical-semantic processing in PPA, in virtue of its involvement in naming performance [58], and semantic error production [49, 59]. While impairment of this region is one of the hallmark of svPPA [60], it appears to be also implied, to a lesser extent, in other PPA variants [3, 61].

The majority of lvPPA patients were represented in a large but circumscribed area, around 3D and 2I profiles. 3D profile coded for the number of verbs and *working memory* errors in sentence repetition, and 2I for *phonology* with errors in non-words repetition and *working memory* errors in sentence comprehension, identifying all features characterizing this variant. These results plausibly reconcile the ambiguities reported in literature, by confirming the solid entity of the logopenic variant [3], but also revealing its relative heterogeneity in language impairments. All lvPPA shared lexical and verbal WM deficits, which was assessed by sentence repetition or comprehension task. Although sentence repetition deficit is considered a core feature for diagnosing lvPPA [1], sentence comprehension was not reported as cardinal criteria, despite being generally impaired [2, 3]. Accordingly, patients belonging to 2I, despite having a lower WM score on sentence comprehension, also reported low WM scores on sentence repetition (see Supplementary Table 2, Additional File 1).

WM errors in sentence comprehension were predicted by the metabolism in the middle/superior frontal gyrus, inferior parietal lobule, and posterior MTG. While the role of fronto-parietal and inferior parietal regions in phonological and sequence manipulation is well established [14, 62, 63], the role of the posterior MTG is more debated. Some proposals suggest that the posterior MTG/STG contributes to phonological WM (see above), due to its connection with frontal regions [64]. Others considered it as a hub for lexico-syntactic elaboration, pivotal for sentence comprehension [65]. In PPA patients, hypometabolism in these areas has been associated with increased WM errors in sentence comprehension and repetition [24].

Supporting the heterogeneity within the logopenic variant, some patients showed a combination of lexical and WM deficits, with phonological impairment in non-word repetition, and/or marked lexical deficit for verbs production, which was previously reported in lvPPA, and interpreted as retrieval deficit [66]. Despite the fact that non-word repetition could reveal difficulties at the phonological level, as items cannot benefit of lexical-semantic reactivation [67, 68], this task was rarely used, likely because it was not reported in the first systematic descriptions of lvPPA [2, 69]. Usually, lvPPA performed worse than controls in this task [68, 70] (but see [71]), with phonological errors being the most frequent error type [68]. Phonological errors in non-word repetition correlated with metabolism in the posterior MTG and in the anterior fusiform gyrus. The posterior temporal lobe is a core component of the phonological loop [72] and is strictly related to phonological errors [73]. It is noteworthy that a recent study found decreased beta-band neural activity in lvPPA patients in the temporal lobe. This reduction was associated with auditory encoding and may reflect the typical phonological processing deficit of these patients [73].

2I and 3D profiles included other patients than lvPPA; lvPPA + populated both profiles, while svPPA only the 3D. The 3D profile was the most heterogeneous one, including all variants, suggesting that some typical, but not cardinal, logopenic features are common in patients not clearly classified by the current guidelines. svPPA patients belonging to the 3D profile showed prominent lexico-retrieval impairment, especially for verbs, and a WM deficit in sentence repetition. These patients displayed also mild semantic impairment, with reduced performance in naming and association tasks. Deficits in WM task, as digit span [12], and in sentence repetition have been already described in svPPA [24, 74]. Patients may fail in retrieval as a result of the semantic deficit, which hinders their ability to use proper strategies to recall target words [75]. It may be speculated that in svPPA the impaired subsystem of WM was the semantic, rather than the phonological one [76]. Comparing svPPA in 3D and 2D profiles, we did not find differences in age, education level, or disease duration, but see [77], highlighting the need to further explore the relationship between genotype/pathology and clinical presentation.

In between 2D and 2I, the 3I profile was characterized by phonological and semantic impairments, and almost exclusively populated by lvPPA + , with no lvPPA included in this profile. lvPPA + is a recent entity and its definition remains highly uncertain, since patients meet logopenic diagnostic criteria, and have additional features but do not fulfill the “mixed” criteria. Considering differences in terms of disease progression, lvPPA + had a significantly longer disease duration than lvPPA, which can be a confounding factor in the definition and characterization of lvPPA + entity. It is indeed extremely difficult to discriminate between the effect of pathological progression and the temporal manifestation of different linguistic deficits. We can hypothesize that phonologic and WM impairments characterize the first stage of the disease, and that semantic difficulties could emerge later on. These results are consistent with longitudinal studies on PPA [78], where lvPPA frequently develops semantic deficit (see PPA-extended [78]), and is in line with the description of advanced cases [67]. The 3I profile should not be interpreted as a profile clearly distinct from the others populated by lvPPA patients, but as a space on the continuum characterized by a combination of spared and impaired language abilities, and into which lvPPA patients from other profiles may move with the disease progression.

Mixed PPA did not cover a precise localization [24, 61], mimicking the multifaced presentation of this clinical manifestation. Mixed PPA were included in all combinations of profiles, except for 2D. Their linguistic profile was similar to the one of lvPPA, but the small sample size prevented us from drawing strong conclusions. Difficulties in conceptualizing the nature of linguistic impairment of mixed patients have been recently addressed [79], but the possibility of identifying the main impaired/spared abilities of each subject may help to reduce unclassifiable cases.

### Limitations

The small sample size of the mixed PPA group prevented us from a clear characterization of these patients. In addition, the lack of biomarkers and pathological data prevents us from drawing an exhaustive characterization of all PPA patients. Further studies should be performed to fill these gaps.

## Conclusion

In conclusion, a new multidimensional approach allowed us to describe the complexity of the linguistic performance of PPA patients, and to visualize the continuum of impairment across logopenic and semantic variants. We identified seven clinical markers that clinicians might use to define impaired/spared abilities and to stage the disease progression and consequent erosion of the language network. This is one of the first attempts to describe the distribution of lexico-semantics, phonology, and WM impairments across the logopenic and semantic variants. The findings could enhance language organization’s comprehension, leading to improved diagnosis and treatment of patients.

### Supplementary Information

**Additional file 1.** Additional file includes Supplementary Table 1, which shows the performance of PPA patients in SAND battery tasks; Supplementary Table 2, which shows comparison of demographic and linguistic significant features emerged from PAMS analysis of the main combinations of profiles; and Supplementary Table 3, which shows demographic features of patients falling in unexpected profiles.

## Data Availability

The datasets used and/or analyzed during the current study are available from the corresponding author on reasonable request.

## Abbreviations

1D: First profile
2D: Second direct profile
2I: Second inverse profile
3D: Third direct profile
3I: Third inverse profile
FDG-PET: Fluorodeoxyglucose (18F) Positron Emission Tomography
GRN: Progranulin gene
HC: Healthy controls
lvPPA: Logopenic variant of primary progressive aphasia
lvPPA +: Logopenic variant of primary progressive aphasia with additional symptoms
MMSE: Mini-Mental State Examination
MTG: Middle temporal gyrus
nf/avPPA: Non-fluent/agrammatic variant of primary progressive aphasia
PAMS: Profile Analysis based on Multidimensional Scaling
PD: Picture description
PHON: Phonological
PL: Parietal lobule
PPA: Primary progressive aphasia
ROI: Region of interest
SAND: Screening for Aphasia in NeuroDegeneration
SEM: Semantic
STG: Superior temporal gyrus
svPPA: Semantic variant of primary progressive aphasia
WM: Working memory
