# Supplementary material for: Heterogeneity and overlap in the continuum of linguistic profile of logopenic and semantic variants of primary progressive aphasia: a Profile Analysis based on Multidimensional Scaling study
Source: Alzheimers Res Ther. 2024 Mar 7;16:49. doi: 10.1186/s13195-024-01403-0 (PMC10918940; doi:10.1186/s13195-024-01403-0)
Supplement: Supplementary file 1 — Additional file 1. Additional file includes Supplementary Table 1, which shows the performance of PPA patients in SAND battery tasks; Supplementary Table 2, which shows comparison of demographic and linguistic significant features emerged from PAMS analysis of the main combinations of profiles; and Supplementary Table 3, which shows demographic features of patients falling in unexpected profiles. [file 13195_2024_1403_MOESM1_ESM.docx]

**Neuropsychological assessment**

All patients underwent a detailed neuropsychological assessment evaluating:

- verbal episodic memory, using the delayed recall of the Rey Auditory Verbal Lerning (1);
- visuo-spatial episodic memory, using the recall of copy of the Rey-Osterrieth figure (2);
- verbal and visuo-spatial short term memory, using the Digit Span Forward and the Corsi Span Forward, respectively (3);
- attentive and executive functions, using the Stroop test (4), the Trial Making Test (5), phonemic fluency (6), and Digit Span Backwards (3);
- visuo-constructional functions, using the copy of the Rey-Osterrieth complex figure (2);
- praxis, using the test of Imitatons of Gestures (7), and the test of bucco-facial apraxia (8);
- language, using the semantic fluency task (6), the naming subtest of the CaGi battery (9), the Token Test (10), and SAND battery (11).

Subject’s performance obtained in each neuropsychological task was corrected according to the respective normative data. Mean performance (calculated on the corrected scores), standard deviation and percentage of impaired performance for each group, namely svPPA, lvPPA, lvPPA+ and mixed PPA variants, are reported in table 1.

|  | **svPPA** | | **lvPPA** | | **lvPPA+** | | **mixed PPA** | |
| --- | --- | --- | --- | --- | --- | --- | --- | --- |
|  | **mean (sd)** | **% impaired** | **mean (sd)** | **% impaired** | **mean (sd)** | **% impaired** | **mean (sd)** | **% impaired** |
| Rey Auditory Verbal Learning |  |  |  |  |  |  |  |  |
| Immediate recall (cut-off < 28.52) | 26.05 (8.11) | 57.14 | 22.73 (8.35) | 76.92 | 20.67 (6.59) | 83.33 | 21.56 (6.93) | 66.67 |
| Delayed recall (cut-off < 4.68) | 3.27 (2.28) | 64.29 | 2.25 (2.87) | 76.92 | 3.85 (1.97) | 75.00 | 1.56 (1.84) | 100.00 |
| Recall of Rey-Osterrieth figure (cut-off <9.46) | 10.6 (6.97) | 40.00 | 7.55 (6.30) | 50.00 | 7.11 (6.11) | 55.56 | 8.66 (4.04) | 66.67 |
| Digit Span Forward (cut-off <4.26) | 5.41 (1.18) | 17.65 | 4.44 (0.92) | 36.84 | 3.93 (0.85) | 58.82 | 4.62 (0.68) | 50.00 |
| Corsi Span Forward (cut-off <3.46) | 5.10 (0.88) | 5.88 | 5.91 (6.46) | 21.43 | 3.90 (1.03) | 27.27 | 4.77 (0.94) | 0.00 |
| Stroop test |  |  |  |  |  |  |  |  |
| Error (cut-off >4.23) | 3.09 (8.74) | 7.69 | 5.12 (6.41) | 30.00 | 5.25 (5.78) | 50.00 | 16.33 (20.88) | 66.67 |
| Time (cut-off >36.91) | 22.59 (17.31) | 23.08 | 45.05 (27.27) | 40.00 | 53.31 (23.72) | 75.00 | 22.00 (19.50) | 33.33 |
| Trial Making Test |  |  |  |  |  |  |  |  |
| A (cut-off >94) | 37.35 (21.02) | 0.00 | 57.35 (63.62) | 14.29 | 117.37 (71.99) | 50.00 | 46.5 (13.43) | 0.00 |
| B (cut-off >283) | 147.36 (100.81) | 9.09 | 166.25 (113.22) | 0.00 | 205.5 (286.37) | 50.00 | 136 (28.28) | 0.00 |
| B-A (cut-off >187) | 112.72 (86.49) | 18.18 | 129.37 (91.77) | 12.50 | 143 (202.23) | 50.00 | 89.5 (14.4) | 0.00 |
| Phonemic fluency (cut-off <16) | 21.25 (8.12) | 29.41 | 22.71 (10.26) | 33.33 | 10.78 (7.44) | 80.00 | 19.46 (5.46) | 66.67 |
| Digit Span Backward (cut-off <2.65) | 3.92 (1.17) | 12.50 | 2.84 (0.78) | 46.67 | 2.71 (0.45) | 33.33 | 3.08 (0.39) | 0.00 |
| Copy of Rey-Osterrieth complex figure (cut-off <28.87) | 31.88 (4.07) | 20.00 | 37.67 (7.04) | 42.86 | 17.91 (11.87) | 66.67 | 34 (1.75) | 0.00 |
| Imitation of Gestures | 69.63 (3.72) | 0.00 | 68.00 (3.16) | 0.00 | 56.44 (13.72) | 44.44 | 70.33 (1.52) | 0.00 |
| Bucco-facial apraxia (cut-off >17) | 19.75 (0.70) | 0.00 | 19.71 (0.75) | 14.29 | 18.06 (1.63) | 25.00 | 18.66 (1.15) | 0.00 |
| Semantic fluency (cut-off <24) | 17.18 (9.89) | 76.47 | 23.55 (8.74) | 53.33 | 14.6 (11.19) | 90.00 | 23 (9.53) | 33.33 |
| CaGi (cut-off <41.48) | 28.26 (15.60) | 84.62 | 41.30 (4.28) | 57.14 | 33.90 (9.01) | 72.73 | 43.54 (1.02) | 0.00 |
| Token Test (cut-off <26.25) | 26.63 (6.93) | 30.77 | 26.73 (5.09) | 40.00 | 19.56 (7.35) | 90.91 | 29.00 (2.04) | 0.00 |
| **SAND** | | | | | | | | |
| Naming (cut-off: ≤9.969) | 6.974 (4.794) | 70.00 | 5.159 (4.189) | 84.21 | 5.838 (4.673) | 73.91 | 10.314 (3.153) | 40.00 |
| Sentence comprehension (cut-off: ≤6.157) | 6.002 (2.05) | 47.37 | 6.756 (1.306) | 38.89 | 5.84 (2.052) | 56.52 | 5.792 (1.881) | 60.00 |
| Word comprehension (cut-off: ≤10.258) | 8.454 (3.412) | 57.89 | 9.887 (2.799) | 42.11 | 8.984 (3.243) | 60.87 | 9.158 (1.526) | 80.00 |
| Repetition TOT (cut-off: ≤6.349) | 7.582 (1.9) | 20.00 | 7.892 (1.679) | 22.22 | 7.313 (1.855) | 34.78 | 7.684 (1.777) | 40.00 |
| Word Repetition (cut-off: ≤4.928) | 5.614 (1.121) | 5.00 | 5.897 (0.462) | 5.56 | 5.668 (0.76) | 8.70 | 5.619 (0.879) | 20.00 |
| Non word repetition (cut-off: ≤0.483) | 1.865 (1.403) | 25.00 | 1.855 (1.584) | 22.22 | 1.778 (1.544) | 30.43 | 2.012 (1.593) | 20.00 |
| Sentence Repetition (cut-off: ≤2.455) | 3.082 (1.865) | 55.00 | 2.211 (1.653) | 72.22 | 1.892 (1.499) | 78.26 | 2.525 (1.431) | 60.00 |
| Reading TOT (cut-off: ≤13.489) | 14.187 (2.618) | 20.00 | 13.974 (3.205) | 26.32 | 14.84 (1.768) | 21.74 | 14.676 (2.097) | 20.00 |
| Word Reading (cut-off: ≤10.106) | 10.707 (2.111) | 20.00 | 10.463 (2.503) | 26.32 | 11.129 (1.391) | 17.39 | 11.642 (0.86) | 20.00 |
| Non word Reading (cut-off: ≤2.228) | 3.488 (0.729) | 5.00 | 3.521 (0.933) | 10.53 | 3.727 (0.561) | 4.35 | 3.237 (1.261) | 20.00 |
| Writing UI (cut-off: ≤2.132) | 2.978 (2.015) | 33.33 | 3.333 (1.951) | 23.53 | 2.86 (1.855) | 31.82 | 3.543 (1.485) | 20.00 |
| Writing Number of word (cut-off: ≤5.908) | 15.224 (10.843) | 22.22 | 17.324 (10.706) | 11.76 | 14.768 (13.499) | 18.18 | 16.385 (8.864) | 20.00 |
| Writing Nouns/Number total words (cut-off: ≤0.15) | 0.214 (0.16) | 27.78 | 0.227 (0.083) | 12.50 | 0.201 (0.099) | 13.64 | 0.242 (0.08) | 20.00 |
| Writing verbs/Number total word (cut-off: ≤0.105) | 0.177 (0.118) | 22.22 | 0.243 (0.096) | 0.00 | 0.245 (0.19) | 0.00 | 0.227 (0.125) | 20.00 |
| Writing number correct syntactic structures/total number of syntactic structures (cut-off: ≤0.75) | 0.578 (0.455) | 44.44 | 0.755 (0.388) | 25.00 | 0.685 (0.429) | 36.36 | 0.7 (0.447) | 20.00 |
| Writing number of orthographic errors (cut-off≥4.776) | 1.176 (1.907) | 22.22 | 1.897 (2.463) | 6.25 | 1.229 (1.93) | 13.64 | 1.485 (1.794) | 20.00 |
| Writing number of lexico-semantic errors/number of words (cut-off: ≥0.033) | 0.021 (0.055) | 27.78 | 0.069 (0.124) | 31.25 | 0.103 (0.224) | 40.91 | 0.071 (0.088) | 40.00 |
| Semantic Association (cut-off: ≤1.166) | 2.398 (1.158) | 16.67 | 2.453 (1.235) | 17.65 | 2.236 (1.306) | 22.73 | 1.596 (0.887) | 40.00 |
| Picture Description_SummerTimeUI (cut-off: ≤3) | 5.15 (2.134) | 25.00 | 4.793 (2.109) | 22.22 | 4.957 (2.078) | 26.09 | 4.8 (2.28) | 20.00 |
| Picture Description_SummerTime_Number of word (cut-off: ≤42.428) | 116.88 (45.636) | 0.00 | 133.054 (70.556) | 11.11 | 107.467 (50.523) | 4.35 | 127.311 (55.344) | 0.00 |
| Picture Description_ SummerTime_Number of nouns/total number of words (cut-off: ≤0.198) | 0.223 (0.043) | 25.00 | 0.206 (0.068) | 55.56 | 0.19 (0.062) | 60.87 | 0.199 (0.041) | 60.00 |
| Picture Description_SummerTime_Number of verbs/total number of words (cut-off: ≤0.115) | 0.182 (0.045) | 5.00 | 0.177 (0.036) | 5.56 | 0.196 (0.039) | 0.00 | 0.194 (0.029) | 0.00 |
| Picture Description_SummerTime_Number of sentences (cut-off: ≤3.553) | 12.443 (5.742) | 5.00 | 12.761 (8.036) | 16.67 | 10.52 (4.646) | 4.35 | 14.047 (10.324) | 0.00 |
| Picture Description_SummerTime_Number of subordinates/total number of sentences (cut-off:0) | 0.495 (0.528) | 0.00 | 0.338 (0.252) | 11.11 | 0.347 (0.276) | 8.70 | 0.277 (0.108) | 0.00 |
| Picture Description_SummerTime_Number of repaired sequences/total number of words (cut-off: ≥0.121) | 0.072 (0.055) | 20.00 | 0.063 (0.054) | 11.11 | 0.093 (0.062) | 17.39 | 0.025 (0.022) | 0.00 |
| Picture Description_SummerTime_Number of phonological errors/total number of words (cut-off: ≥0.0186) | 0.009 (0.018) | 20.00 | 0.014 (0.031) | 16.67 | 0.013 (0.021) | 17.39 | 0.027 (0.061) | 20.00 |
| Picture Description_SummerTime_Number of semantic errors/total number of words (cut-off: ≥0.035) | 0.034 (0.021) | 50.00 | 0.049 (0.043) | 61.11 | 0.046 (0.038) | 47.83 | 0.048 (0.052) | 60.00 |

Table 1 - Mean, standard deviation and percentage of impaired cases for each task for each PPA group.

**Demographic and linguistic variables between core profiles.**

Subjects belonging to the four core profiles (2D, 2I, 3D, 3I) were compared in terms of the demographic variables and of scores obtained to the tasks emerged as significant from the PAMS analysis. A Kruskal Wallis test, and post hoc corrected using Bonferroni (0.05/4=0.012) were carried out.

| **Core profiles** | | | | | | |
| --- | --- | --- | --- | --- | --- | --- |
|  | **2D**  **(n=14)** | **2I**  **(n=16)** | **3D**  **(n=18)** | **3I**  **(n=15)** | **P value** | **Post hoc** |
| *Demographics* | | | | | | |
| Age (years) | 70.21 | 71.06 | 66.05 | 71.33 | 0.216 | - |
| Education (years) | 10.57 | 13.06 | 11.72 | 10.47 | 0.421 | - |
| disease duration (months) | 30.5 | 24.46 | 24 | 32.64 | 0.17 | - |
| MMSE PG | 21.3 | 21.8 | 20.92 | 20.33 | 0.914 | - |
| *Significant features from PAMS* | | | | | | |
| PD N Nouns | 0.198 | 0.205 | 0.215 | 0.199 | 0.908 | - |
| PD N verbs | 0.21 | 0.175 | 0.173 | 0.193 | 0.048 | - |
| SEM Naming | 0.168 | 0.705 | 0.564 | 0.429 | <0.001 | 2D < 2I, 3D; 3I < 2I |
| SEM semantic association | 0.357 | 0.797 | 0.819 | 0.383 | <0.001 | 2D < 2I, 3D;  3I < 2I, 3D1 |
| PHON Non words repetition | 0.679 | 0.109 | 0.764 | 0.183 | <0.001 | 2I < 2D, 3D; 3I < 2D, 3D |
| WM sentence repetition | 0.779 | 0.597 | 0.643 | 0.67 | 0.288 | - |
| WM sentence comprehension | 0.821 | 0.703 | 0.861 | 0.533 | 0.002 | 2I < 3D;  3I < 2D, 3D |

Table 2 - Comparison between core profiles in terms of demographics and significant PAMS features. SEM: semantic errors; PHON: phonological errors; WM working memory errors.

**Demographic variables of patients belonging to unexpected core profiles.**

Single patients belonging to unexpected core profiles were compared in terms of the demographic variables (age, education and disease duration) to patients of the respective a-priori variant using the method described by Crawford and Garthwaite (2002) (12). In addition, 5 svPPA patients falling in the 3D profile were compared with the other svPPA patients of the 2D profile using the Mann-Whitney test, showing no differences.

|  | **Unexpected**  **(variant, profile)** | **Expected**  **(variant, profile)** |  | **Unexpected**  **(variant, profile)** | **Expected**  **(variant, profile)** |  |
| --- | --- | --- | --- | --- | --- | --- |
|  | svPPA, 3I | svPPA, 2D (n=12) | p value | svPPA, 2D+3D | svPPA, 2D (n=12) | p value |
| age (years) | 76 | 69.66 (6.19) | 0.346 | 66 | 69.66 (6.19) | 0.581 |
| education (years) | 17 | 10.50 (4.66) | 0.207 | 13 | 10.50 (4.66) | 0.616 |
| disease duration (months) | 24 | 29.58 (18.29) | 0.774 | 24 | 29.58 (18.29) | 0.774 |
|  | | | | | | |
|  | **Unexpected**  **(variant, profile)** | **Expected**  **(variant, profile)** |  | **Unexpected**  **(variant, profile)** | **Expected**  **(variant, profile)** |  |
|  | lvPPA, 2D | lvPPA, 2I and 3D (n=19) | p value | lvPPA+, 2D | lvPPA 3I (n=13) | p value |
| age (years) | 65 | 68.36 (6.13) | 0.6 | 82 | 71.15 (8.42) | 0.238 |
| education (years) | 5 | 11.10 (3.19) | 0.079 | 17 | 9.53 (4.99) | 0.175 |
| disease duration (months) | 48 | 20.63 (8.64) | 0.006 | 24 | 34.08 (13.59) | 0.488 |
|  | | | | | | |
|  | **Unexpected**  **(variant, profile)** | **Expected**  **(variant, profile)** |  |  |  |  |
|  | svPPA, 2D  (n=5) | svPPA, 2D (n=12) | p value |  |  |  |
| age (years) | 63.40 (11.76) | 69.66 (6.19) | 0.290 |  |  |  |
| education (years) | 12.20 (4.38) | 10.50 (4.66) | 0.477 |  |  |  |
| disease duration (months) | 28.80 (13.68) | 29.58 (18.29) | 0.780 |  |  |  |

Table 3 – Characterization of cases falling in an unexpected profile. SvPPA= semantic variant of primary progressive aphasia; lvPPA= logopenic variant of primary progressive aphasia meeting canonical criteria; lvPPA+= logopenic variant of Primary Progressive aphasia with additional symptoms; 2D: first and direct second profiles; 2I: first and indirect second profiles; 3D: first and direct third profiles; 3I: first and indirect third profiles; 2D+3D: direct second and direct third profiles. In expected core profiles the values of mean and standard deviation of the group are reported.

**References**

1. Carlesimo GA, Sabbadini M, Fadda L, Caltagirone C. Different Components in Word-List Forgetting of Pure Amnesics, Degenerative Demented and Healthy Subjects. Cortex. 1995;31(4):735-745. doi: 10.1016/S0010-9452(13)80024-X
2. Caffarra P, Vezzadini G, Dieci F, Zonato F, Venneri A. Rey-Osterrieth complex figure: normative values in an Italian population sample. Neurol Sci. 2002;22(6):443-447. doi:10.1007/s100720200003
3. Monaco M, Costa A, Caltagirone C, Carlesimo GA. Forward and backward span for verbal and visuo-spatial data: standardization and normative data from an Italian adult population. Neurol Sci Off J Ital Neurol Soc Ital Soc Clin Neurophysiol. 2013;34(5):749-754. doi:10.1007/s10072-012-1130-x
4. Caffarra P, Vezzadini G, Dieci F, Zonato F, Venneri A. Una versione abbreviata del test di Stroop: Dati normativi nella popolazione Italiana. Nuova Rivista di Neurologia. 2002 Jul;12(4):111-115.
5. Giovagnoli AR, Del Pesce M, Mascheroni S, Simoncelli M, Laiacona M, Capitani E. Trail making test: normative values from 287 normal adult controls. Ital J Neurol Sci. 1996;17(4):305-309. doi:10.1007/BF01997792
6. Novelli G, Papagno C, Capitani E, Laiacona M, al et. Tre test clinici di ricerca e produzione lessicale. Taratura su sogetti normali. [Three clinical tests to research and rate the lexical performance of normal subjects.]. Arch Psicol Neurol Psichiatr. 1986;47(4):477-506.
7. De Renzi E, Motti F, Nichelli P. Imitating Gestures: A Quantitative Approach to Ideomotor Apraxia. Arch Neurol. 1980;37(1):6-10. doi:10.1001/archneur.1980.00500500036003
8. Spinnler H, Tognoni G Standardizzazione e taratura italiana di test neuropsicologici. Ital J Neurol Sci 1987 suppl 8 to 6: 1–120
9. Catricalà E, Della Rosa PA, Ginex V, Mussetti Z, Plebani V, Cappa SF. An Italian battery for the assessment of semantic memory disorders. Neurol Sci. 2013;34(6):985-993. doi:10.1007/s10072-012-1181-z
10. De Renzi E, Vignolo LA. The token test: A sensitive test to detect receptive disturbances in aphasics. Brain. 1962;85:665-678. doi:10.1093/brain/85.4.665
11. Catricalà E, Gobbi E, Battista P, et al. SAND: a Screening for Aphasia in NeuroDegeneration. Development and normative data. Neurol Sci. 2017;38(8):1469-1483. doi:10.1007/s10072-017-3001-y
12. Crawford, J.R., & Garthwaite, P.H. Investigation of the single case in neuropsychology: Confidence limits on the abnormality of test scores and test score differences. Neuropsychologia, 2002; 40, 1196-1208.
